# Preoperative Immunocyte-Derived Ratios Predict Postoperative Recovery of Gastrointestinal Motility after Colorectal Cancer Surgery

**DOI:** 10.3390/jcm12196338

**Published:** 2023-10-03

**Authors:** Andreea Firut, Dragos Nicolae Margaritescu, Adina Turcu-Stiolica, Marius Bica, Ionela Rotaru, Ana-Maria Patrascu, Razvan Ilie Radu, Daniela Marinescu, Stefan Patrascu, Costin Teodor Streba, Valeriu Surlin

**Affiliations:** 1 Department of Surgery, University of Medicine and Pharmacy of Craiova, 200349 Craiova, Romania; maria.firut@umfcv.ro (A.F.); dragos.margaritescu@umfcv.ro (D.N.M.); marius.bica@umfcv.ro (M.B.); valeriu.surlin@umfcv.ro (V.S.); 2 Pharmacoeconomics and Statistical Analysis in Clinical Trials and Pharmaceutical Research, University of Medicine and Pharmacy of Craiova, 200349 Craiova, Romania; adina.turcu@umfcv.ro; 3 Department of Hematology, University of Medicine and Pharmacy of Craiova, 200349 Craiova, Romania; ionela.rotaru@umfcv.ro (I.R.); anamaria.patrascu@umfcv.ro (A.-M.P.); 4 Department of Interventional Cardiology, Prof. Dr. C. C. Iliescu Emergency Institute for Cardiovascular Diseases, 022328 Bucharest, Romania; razvan-ilie.radu@umfcd.ro; 5 Department of Pulmonology, University of Medicine and Pharmacy of Craiova, 200349 Craiova, Romania; costin.streba@umfcv.ro

**Keywords:** ileus, colorectal neoplasms, inflammation, bowel function

## Abstract

The aim of this study was to assess the role of immunocyte-derived ratios (IDRs), such as the systemic immune-inflammation index (SII), neutrophil-to-lymphocyte ratio (NLR), platelet-to-lymphocyte ratio (PLR), and lymphocyte-to-monocyte ratio (LMR), as markers for the postoperative recovery of gastrointestinal function following colorectal cancer surgery. A retrospective analysis was conducted on a consecutive cohort of 260 patients who underwent radical colorectal cancer surgery within the timeframe spanning from January 2016 to December 2022. Data concerning the postoperative recovery of gastrointestinal function included the I-FEED score, time to pass flatus, toleration for liquids in the first 48 h, and the need for nasogastric tube reinsertion in the immediate postoperative period. A special emphasis was allocated towards the examination of IDRs and their interrelation with the postoperative gastrointestinal functional parameters. The I-FEED score exhibited a positive correlation with the NLR, SII, and PLR. The univariate analysis indicated that all IDRs, multiorgan resection, hemoglobin and protein levels, regional nodal extent of the tumor (N), and obesity significantly affected nasogastric tube reinsertion. The multivariate analysis showed that the SII and N1 stages were risk factors for nasogastric tube reinsertion after colorectal cancer surgery. The SII and multiorgan resection were the only classifiers that remained significant in the multivariable analysis for the toleration for liquids. In summation, certain preoperative IDRs, such as the SII, PLR, and NLR, may hold potential as predictive determinants for postoperative gastrointestinal functional recovery following colorectal cancer surgery.

## 1. Introduction

During the last decades, colorectal cancer (CRC) has become a global medical burden, displaying a worrisome increase in incidence and mortality compared to other digestive cancers [1]. Although a vast array of oncologic treatment options is available today, surgery remains the mainstay of treatment for most CRC variants. Nonetheless, surgery for CRC, whether performed through conventional open approaches or minimally invasive methods, shares its burden of complications and adverse events.

Postoperative gastrointestinal motility dysfunction, clinically translated as postoperative ileus (POI), is a common phenomenon following colorectal cancer surgery, bearing detrimental clinical implications and a substantial financial burden [2,3,4]. Both clinical entities are loosely defined terms covering a vast area of early postoperative events with varying definitions, making them difficult to properly analyze [5]. The I-FEED scoring system aims to evaluate the construct of POI in elective colorectal surgery based on five clinical parameters, such as oral intake, nausea, emesis, physical examination, and duration of symptoms. This score, which categorizes the postoperative GI functional impairment into three classes: normal postoperative bowel function, postoperative gastrointestinal (GI) intolerance (POGI), and postoperative GI dysfunction (POGD), is a more comprehensive tool to assess and standardize the postoperative recovery of bowel function [6].

Numerous attempts have been made to treat this condition, but it is increasingly clear that one potential solution to properly manage it is by predicting the risk of occurrence with an adequate level of certainty and prevent it by addressing its contributing factors [7,8,9]. Endogenous factors such as age, certain chronic medication use, smoking, metabolic status, and gut microbiota, as well as exogenous ones such as blood loss, operation time, type of anesthesia, etc., have been proposed as determinants for the postoperative recovery of bowel function [10,11,12]. From a more comprehensive etiopathogenic perspective, ileus can be triggered through three main categories of mechanisms: pharmacologic, inflammatory, and neurogenic. The inflammatory status is of particular interest, as chronic inflammation and acute infections are known to interfere with the postoperative pathophysiological mechanisms of functional bowel recovery and the normal healing process [13,14]. The current body of literature concerning the significance of a preoperative evaluation of inflammation-related biomarkers remains constrained. This pertains to a spectrum of indicators, ranging from basic immunocyte-derived ratios (IDRs) to more intricate indicators such as interleukins, phosphatidylcholine derivatives, galectin-3, or osteopontin, and their potential utility in prognosticating early postoperative convalescence and functional achievements within the context of colorectal surgical procedures [15,16,17]. Amongst the array of potential markers under consideration, IDRs stand out with particular significance, attributed to their cost-effectiveness and simplified integration within the clinical setting. Certain IDRs, such as the NLR and SII, serve as indicators that portray the intricate and dynamic interplay between the innate immune response constituents, primarily neutrophils, and the adaptive cellular immune components, principally lymphocytes, in the context of diverse pathological conditions [18,19]. Furthermore, it is noteworthy that the I-FEED scoring system lacks consistent clinical validation, thereby accentuating the necessity for the provision of validity substantiation through rigorous clinical investigations [20].

Based on these premises, our study aimed to evaluate the potential markers for the postoperative recovery of bowel function, with a special emphasis on IDRs such as the systemic immune-inflammation index (SII), neutrophil-to-lymphocyte ratio (NLR), platelet-to-lymphocyte ratio (PLR), and lymphocyte-to-monocyte ratio (LMR).

## 2. Materials and Methods

### 2.1. Population and Design

This retrospective study was conducted at a tertiary surgical center in Southwest Romania, involving patients who underwent radical surgical procedures for colorectal cancer between January 2016 and December 2022. We classified a surgical intervention as radical when it involved an R0 excision of a colonic tumor, accompanied by central vascular ligation of the vascular pedicles and complete mesocolic excision. In instances of rectal cancer, this encompassed a high tie of the inferior mesenteric artery and ligation of the inferior mesenteric vein at the inferior border of the pancreas, followed by the circumferential dissection of the rectum along the anatomical plane. A macroscopic evaluation of the specimen quality with respect to a complete mesorectal excision was conducted as the standard practice. The study followed patients for a minimum of 2 weeks after their surgery. Inclusion criteria consisted of the histopathologic confirmation of colorectal malignancy, ages above 18 years, and the performance of radical surgery during the hospitalization period. Exclusion criteria comprised all patients who underwent reintervention during the same admission, those with known inflammatory or hematological diseases that may have interfered with the complete blood count, and cases with residual local tumors after surgery or unresectable distant metastases. Notably, transanal endoscopic procedures were also excluded from the analysis.

### 2.2. Surgical Intervention and Perioperative Care

Mechanical bowel preparation with a 4 L polyethylene glycol solution was administered to all cases of rectal surgery, while, in colonic surgery, in cases with a laparoscopic approach, started 48 h before surgery. Antibiotic prophylaxis involved three doses of oral metronidazole (500 mg) administered for two days prior to surgery, along with a single dose of intravenous second-generation cephalosporin during anesthesia induction. Patients requiring anticoagulant therapy received low-molecular-weight heparins (LMWH) until 12 h before surgery, which were then resumed after the procedure with the dosage adjusted based on the patient’s weight and comorbidities. The ERAS protocol was partially followed. Components derived from the ERAS protocol that were implemented included preoperative optimization, anemia screening and management, nutritional care and correction of hyperglycemia, preoperative information and counseling, measures for antiemetic prophylaxis, fasting for at least 4 h for liquids and 6 h for solid foods, maintaining euvolemia, and goal-directed fluid therapy. Radical surgery, either open or laparoscopic, was performed under standard anesthesia protocols, consisting of general anesthesia with endotracheal intubation in all cases. Factors such as tumor location, TNM stage, local vascularization, associated medical or surgical conditions, and anticipated postoperative difficulties were considered when selecting the surgical procedure type and the need for stoma creation. A nasogastric tube was temporarily placed before anesthesia induction and removed at the conclusion of the surgical procedure in all cases. At the end of each surgery, abdominal drains were inserted and typically remained in place until the fluid output decreased to less than 50 mL. Patients were encouraged to ambulate starting from the first postoperative day. Clear liquids were permitted after the initial 24 h following surgery, and a regular diet was gradually reintroduced in the absence of emesis. Opioid analgesics were not used in our therapeutic protocols.

### 2.3. Data Collection

The relevant clinical endpoints, such as the time from the end of surgery to the day of first flatus or fecal discharge; toleration for fluids in the first 48 h after the surgical procedure; nasogastric tube reinsertion during this postoperative interval; and the I-FEED score components (Intake, Feeling nauseated, Emesis, physical Exam, and Duration of symptoms), were recorded in all cases. 

The 2018 I-FEED Scoring System, elaborated by the American Society for Enhanced Recovery After Surgery (ERAS) and Perioperative Quality Initiative Joint Consensus, was used to categorize the postoperative gastrointestinal tract functional impairment. Three I-FEED categories were used: normal (N) for an I-FEED score of 0–2, postoperative gastrointestinal intolerance (POGI) for an I-FEED score of 3–5, and postoperative gastrointestinal dysfunction (POGD) for an I-FEED score of >6. While the first category refers to the normal resumption of intestinal activity following the surgical procedure, the next two categories mirror various degrees of impairment in the process of postoperative gastrointestinal functional healing. The highest daily I-FEED score generated for each patient was recorded in the database and used for the analysis.

All relevant information regarding sex, age, associated pathologic conditions, blood tests, tumor staging, surgical procedures, and details concerning their postoperative recovery were acquired from the hospital’s electronic database. Blood tests such as a complete blood count with the blood differential test and protein levels were obtained in the first 48 h prior to surgery. Each subset of values was analyzed to observe any potential correlations with the main postoperative outcomes: I-FEED score categories, toleration for liquids in the first 48 h (TL), and the need for nasogastric tube reinsertion in the immediate postoperative period (NTR). The time to pass flatus (Tf), defined as the interval (days) from surgery until the first emission of flatus, was independently analyzed.

### 2.4. Statistical Analysis

The data were evaluated for normal distribution using the Kolmogorov–Smirnov test. Continuous variables were presented as the mean ± standard deviation (SD) or median (interquartile range, IQR), while categorical variables were presented as percentages. To assess the differences between continuous variables, the Student’s *t*-test was used for normally distributed variables, and the Mann–Whitney test was used for variables that were not normally distributed. The differences between categorical variables were evaluated using the chi-square test or Fisher’s exact test. In cases where there were more than two groups (e.g., I-FEED score), the Kruskal–Wallis test was employed. All tests were two-sided. For investigating the association of risk factors with the toleration of liquids and NG tube reinsertion, a multivariate logistic regression analysis was conducted. Linear regression analysis was utilized to identify variables that could predict the duration of ileus. A *p*-value less than 0.05 was considered statistically significant. Statistical analysis was performed using GraphPad Prism v.9.5.1 for Windows (GraphPad Software, San Diego, CA, USA. To visually illustrate the correlations between variables, the Pandas df.corr() and Seaborn relplot() functions in Python were used.

## 3. Results

Between 1 January 2015 and 31 December 2022, a total number of 276 cases with colorectal cancer were operated electively in our institution. Sixteen cases were deemed ineligible for inclusion in our study. Specifically, two patients were excluded due to the presence of concurrent malignant hematological conditions. An additional case was omitted from the study due to the occurrence of an acute coronary event during the initial postoperative phase, necessitating immediate endovascular intervention. Furthermore, three cases, consisting of two instances of right colon cancer and one case of rectal cancer, were excluded due to the intraoperative identification of disseminated liver metastases. In two separate cases, the presence of disseminated abdominal disease posed a prohibitive factor against the execution of radical surgical procedures. An additional four patients were excluded owing to inflammatory bowel disease (two instances of Crohn’s disease and two cases of ulcerative colitis). Notably, two cases necessitated prompt reintervention to address postoperative hemorrhage, and an additional case mandated surgical intervention on the third p.o. day due to partial anastomosis dehiscence, subsequently leading to peritonitis. Finally, in one patient with COPD, surgery was performed due to bowel evisceration. The resulting 260 cases were included in the final analysis, with a mean age of 68.3 years and a sex ratio of 1.24. Multiorgan resections were counted in 26 patients (10%). Ninety-one procedures (35% of cases) ended with stoma creation, either temporary or definitive. A laparoscopic approach was used in 43 cases (16.5%), with a conversion rate of 9.3%. Thirty-seven patients (14.23%) experienced vomiting after oral feeding, requiring reinsertion of NGT in 27 cases (10.3%). The clinical and biological characteristics of these patients are summarized in Table 1 and Table 2.

Statistically significant differences were observed for the TL and NGtR groups in terms of IDRs and the need for multiorgan resection, as well as protein levels. However, the SII and multiorgan resection were the only classifiers that remained significant in the multivariable analysis for both TL and NGtR (Table 3 and Table 4).

The univariate analysis proved that the NLR, PLR, LMR, SII, multiorgan resection, Hb, protein, N stage, and obesity significantly affected nasogastric tube reinsertion. The multivariate analysis showed that the SII and N1 stages were risk factors for nasogastric tube reinsertion after colorectal cancer surgery.

A statistically significant decline was shown in the hemoglobin of patients with POGD when compared to normal patients (*p*-value = 0.038). The TNM stage did not influence the I-FEED, as indicated in Table 5.

**Figure 1:**
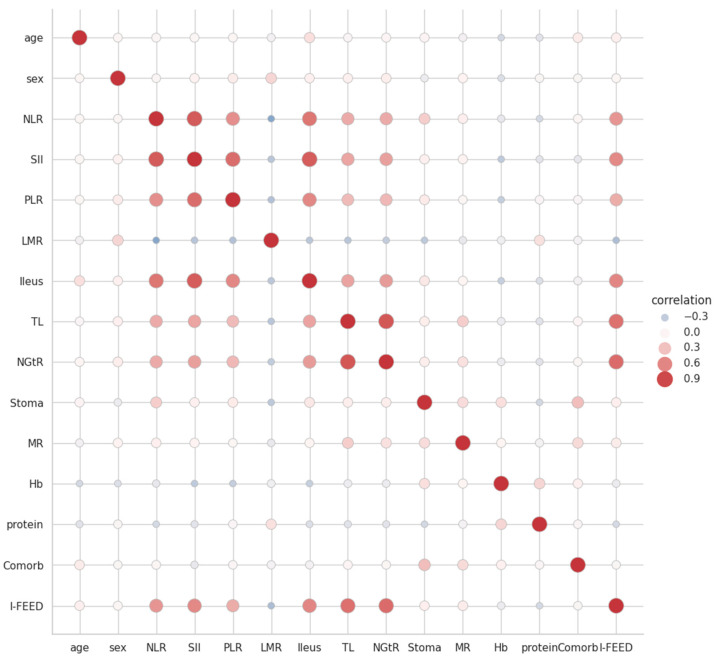
The plot of the correlation matrix for the measured variables. NLR, neutrophil-to-lymphocyte ratio; SII, systemic immune-inflammation index; PLR, platelet-to-lymphocyte ratio; LMR, lymphocyte-to-monocyte ratio; Hb, hemoglobin; TL, toleration for liquids; NGtR, nasogastric tube reinsertion; MR, multiorgan resection; Comorb, number of comorbidities.

Moreover, the analysis indicated a moderate positive correlation between I-FEED and the NLR (rho = 0.523, *p*-value < 0.0001), SII (rho = 0.581, *p*-value < 0.0001), PLR (rho = 0.40, *p*-value < 0.0001), Tf (rho = 0.595, *p*-value < 0.0001), toleration for liquids (rho = 0.709, *p*-value < 0.0001), and NG tube reinsertion (rho = 0.727, *p*-value < 0.0001). A significant moderate negative correlation was present between I-FEED and the LMR (rho = −0.399, *p*-value < 0.0001). Additionally, a significant low negative correlation was discovered between protein and I-FEED (rho = −0.226, *p*-value < 0.0001), indicating lower protein values were found in POGD patients. No correlations were observed between I-FFED and age (rho = 0.059, *p*-value = 0.346), gender (rho = −0.001, *p*-value = 0.992), comorbidities (rho = −0.040, *p*-value = 0.519), Hb (rho = −0.115, *p*-value = 0.064), stoma (rho = 0.065, *p*-value = 0.299), or multiorgan resection (rho = 0.082, *p*-value = 0.186) (Figure 1).

Notably, the duration until the occurrence of the first flatus was observed to be affected by the T stage, whereby patients with T1 or T2 tumors experienced a shorter time compared to those with T3 or T4 tumors (*p*-value = 0.002). Similarly, the N stage was found to have an impact, with N0 patients having a shorter duration compared to N1 and N2 patients (*p*-value = 0.004). However, the M stage did not demonstrate a significant association with the time to first flatus (*p*-value = 0.170). Subsequently, we conducted a search to identify variables that could predict the duration of the intestinal paresis, assessed as Tf. The regression equation, as shown in Formula (1), revealed that certain indicators, such as the PLR and I-FEED, have a positive association, while Hb has a negative association. Our model yielded a statistical R value of 0.764 and an R-squared value of 0.584, indicating the proportion of variance in the response variable, Tf (*p* < 0.0001). According to the linear regression analysis, Tf (days) can be defined by the PLR, SII, Hb, and I-FEED, with the highest weight assigned to I-FEED, as illustrated below:

Tf = 2.13 + 0.806 × I-FEED + 0.003 × PLR − 0.042 × Hb(1)

## 4. Discussion

Our study has identified a comprehensive set of intraoperative and biological factors that appear to have a positive correlation with the delayed resolution of bowel function. IDRs were selected due to their accessibility and widespread usage, as well as their tendency to reflect the inflammatory status of patients. The decision to perform a preoperative analysis of the IDRs was justified by the necessity to identify the patients at risk before undergoing the surgical procedure, enabling the initiation of adequate treatment. Furthermore, the important changes that may appear in blood cell ratios during the surgical procedure, resulting from varying degrees of blood loss and intraoperative transfusions, could influence their significance and the prognostic values of these indicators. Presently, IDRs are extensively utilized across a diverse spectrum of medical disciplines as a dependable and conveniently accessible metric for gauging the immune reaction to a range of both infectious and noninfectious triggers. These ratios are subject to the influences of numerous factors, encompassing variables like diabetes, obesity, psychiatric disorders, solid organ malignancies, sepsis, pharmaceutical interventions, acute and chronic vascular disease, including coronary heart disease and stroke, and viral infections such as the SARS-CoV-2 virus [21,22]. Our analysis suggests that the preoperative values of three of the IDRs—namely, NLR, SII, and PLR, respectively—are elevated in POGD patients and seem to positively correlate with the duration of intestinal paresis, as assessed by the Tf, TL, and NGtR. 

Concerning the need for an independent analysis of various subsets of variables, including those encompassed in the I-FEED score or POI definition, there is a reasonable suspicion of an underlying causal relationship between these entities. This suspicion arises due to their shared emphasis on postoperative disruption in bowel peristalsis. At the same time, individually assessing each symptom provides a more comprehensive overview of the functional changes that appear in the postoperative setting. This approach also facilitates the examination of the degree of interdependence between IDRs and each of these specific variables.

One major limitation in the analysis of POI is the lack of a universally accepted definition concerning this complication. Its reported occurrence varies widely, spanning from 48 h to a week, without a clear consensus on the fundamental parameters for its evaluation. Consequently, this variability influences the accurate determination of both the incidence rate and appropriate management for this postoperative complication. Moreover, the absence of dedicated guidelines specifically addressing this issue further complicates the efforts associated with accurately identifying and treating POI. Our study indicated an incidence of prolonged POI, defined as ileus lasting more than 4 days following surgery, of 8.84%. This finding aligns with similar rates reported by other authors, which range from 5% to 30% [23,24]. 

The exact causes and mechanisms behind the prolonged absence of normal bowel function following a surgical procedure remain not entirely elucidated. Autonomic digestive neuron dysfunction and imbalances in gastrointestinal hormones, especially motilin, ghrelin, and 5-hydroxytryptamine, are recognized as the main factors implicated in this phenomenon. Additionally, the pathophysiology involves other factors, such as postoperative bowel edema, demodulation in the enteric nervous system, and disruption of glial cell activity triggered by mechanical forces, which are direct consequence of the surgical procedure. The postoperative neurodigestive dysfunction process is due to the inhibition of interdigestive migrating motor contractions, especially the low postprandial response, reduced activity in phase II, and interrupted or retrograde phase III, with some peristaltic activity migrating orally [25,26,27]. A novel and intriguing conjecture arises in the form of distal colonic hyperactivity, posited on the observation that, following right hemicolectomy, a marked elevation in cyclic motor patterns occurs within the sigmoid colon [28]. This underlying pathophysiological framework potentially bears significant therapeutic ramifications for postoperative ileus (POI) and postoperative gastrointestinal dysfunction (POGD). This proposition intimates that, in specific instances, the pursuit of augmenting intestinal peristalsis might not be the appropriate therapeutic objective.

If intrinsic factors such as age, ischemia, and other chronic conditions are impossible to correct, there are other potential determinants that could be addressed to reduce the risk of POI and POGD. These include modifying the use of certain volatile gases during general anesthesia, adjusting the complexity of the surgical procedure, or optimizing the postoperative doses of opioids [29]. 

An attractive solution for a more accurate identification of patients at risk for POI would involve the use of readily available blood tests. Markers such as the preoperative albumin levels have shown the potential to influence the restoration of intestinal peristalsis following abdominal surgery [30].

Although extensive effort has been devoted to analyzing the role of IDRs as prognostic determinants pertinent to postoperative morbidity and survival outcomes, only a few studies have specifically explored the significance of these markers as predictors in the context of POI within the sphere of gastrointestinal surgery [31]. 

Our study suggests that elevated levels of specific IDRs, such as the NLR or SII, reflects a proinflammatory state that could impede the normal recovery and healing process of the bowels. However, this role is limited, as our linear regression equation seems to suggest, which could be explained through a multitude of causes. Gastrointestinal postoperative recovery may be influenced by certain comorbidities, the type of anesthesia, perioperative nutritional support, bowel manipulation and surgical trauma, the use of pneumoperitoneum and intraabdominal pressure, postoperative drugs regimens, etc. [32]. Nevertheless, inflammation in itself is a recognized exacerbating factor for postoperative ileus and gastrointestinal healing, so any alteration of the preoperative balance of proinflammatory and anti-inflammatory protective factors should be recognized and properly managed [33]. Various therapeutic strategies have been proposed to address this underlying pathophysiological pathway, ranging from remote ischemic preconditioning to the use of anti-inflammatory agents, with promising results [34,35].

This study has several limitations, mainly due to its retrospective, single-center design. One should interpret the overall results with caution due to population heterogeneity and the relatively small sample size. A larger sample size or prospectively acquired database may offer improved results. Another limitation concerns the insufficient clinical validation of the I-FEED score, considering its relatively recent introduction [34,36,37]. Although this score is an easy-to-use instrument with a potentially reasonable predictive power, there are other scoring algorithms, such as the one suggested by Van Bree et al., based on several clinical parameters and supported by the scintigraphic assessment of bowel transit, which may better predict the resolution of gastrointestinal motility [38].

## 5. Conclusions

Preoperative IDRs seem to predict the postoperative recovery of bowel function through the analysis of some of its most widely used clinical parameters, such as the POI, postoperative toleration for liquids, and the I-FEED score. Other factors, such as the protein blood level and TNM stage, may also play a role in the resumption of normal function of the digestive tract after colorectal surgery.

## Figures and Tables

**Table 1:** The main demographic, clinical, and surgical characteristics of patients with and without toleration to liquids.

Characteristics (Mean ± SD, Median (IQR))	All Patients N = 260	Patients with TL N = 37	Patients without TL N = 223	*p*-Value
Age, years	68.39 ± 10.44 70 (63–76)	68.14 ± 9.3 66 (64–75.5)	68.43 ± 10.63 70 (63–76)	0.6
Sex male, *n* (%)	144 (55.4%)	18 (48.6%)	126 (56.5%)	0.379
NLR	4.3 ± 3.6 3.2 (2.3–4.7)	8.7 ± 5.4 7.6 (4.1–13.3)	3.5 ± 2.6 3.0 (2.2–4.0)	<0.0001 ****
PLR	189.9 ± 101.32 168 (123.5–231.1)	284.4 ± 133.1 246.2 (197.3–355.6)	174.2 ± 85.8 160.6 (118.5–217.8)	<0.0001 ****
LMR	3.9 ± 8.5 2.9 (1.8–4.2)	1.9 ± 1.4 1.5 (1.0–2.6)	4.3 ± 8.8 3 (1.9–4.3)	<0.0001 ****
SII	1254.4 ± 1354.1 862.1 (528.3–1424.4)	3281.9 ± 2450.3 2428.2 (1523.5–4800.1)	918.0 ± 605.7 774.1 (496.8–1253.7)	<0.0001 ****
Stoma, yes (%)	90 (34.6%)	16 (43.2%)	74 (33.2%)	0.264
Multiorgan Resection, yes (%)	26 (10%)	10 (27%)	16 (7.2%)	0.001 **
Hb	10.9 ± 2.4 11 (9.3–12.5)	10.3 ± 2.5 10.6 (8.1–12.1)	11.0 ± 2.4 11.0 (9.6–12.5)	0.097
Protein	6.3 ± 0.9 6.4 (5.8–6.9)	5.9 ± 1.0 5.9 (5.1–6.8)	6.4 ± 0.9 6.4 (5.9–6.9)	0.011 *

SD, standard deviation; IQR, interquartile range; TL, toleration to liquids; NLR, neutrophil-to-lymphocyte ratio; PLR, platelet-to-lymphocyte ratio; LMR, lymphocyte-to-monocyte ratio; SII, systemic immune-inflammation index; Hb, hemoglobin; *, *p*-value < 0.05; **, *p*-value < 0.01; ****, *p*-value < 0.0001 for the statistical significance.

**Table 2:** The main demographic, clinical, and surgical characteristics of patients with and without nasogastric tube reinsertion.

Characteristics (Mean ± SD, Median (IQR))	All Patients N = 260	Patients with NG Tube Reinsertion N = 27	Patients without NG Tube Reinsertion N = 233	*p*-Value
Age <years>	68.39 ± 10.44 70 (63–76)	68.14 ± 9.3 66 (64–75.5)	68.33 ± 10.52 70 (62.5–76)	0.837
Sex male, *n* (%)	144 (55.4%)	12 (44.4%)	132 (56.7%)	0.307
NLR	4.3 ± 3.6 3.2 (2.3–4.7)	8.7 ± 5.4 7.6 (4.1–13.3)	3.7 ± 2.9 2.99 (2.2–4.2)	<0.0001 ****
PLR	189.9 ± 101.32 168 (123.5–231.1)	284.4 ± 133.1 246.2 (197.3–355.6)	175.5 ± 85.6 161.0 (118.6–218.0)	<0.0001 ****
LMR	3.9 ± 8.5 2.9 (1.8–4.2)	1.9 ± 1.4 1.5 (1.0–2.6)	4.2 ± 8.9 3 (1.9–4.3)	<0.0001 ****
SII	1254.4 ± 1354.1 862.1 (528.3–1424.4)	3281.9 ± 2450.3 2428.2 (1523.5–4800.1)	983.7 ± 928.2 774.1 (502.3–1267.7)	<0.0001 ****
Stoma <yes> (%)	90 (34.6%)	12 (44.4%)	78 (33.5%)	0.288
Multiorgan Resection <yes> (%)	26 (10%)	6 (22.2%)	20 (8.6%)	0.038 *
Hb	10.9 ± 2.4 11 (9.3–12.5)	10.3 ± 2.5 10.6 (8.1–12.1)	11.0 ± 2.4 10.98 (9.6–12.5)	0.071
Protein	6.3 ± 0.9 6.4 (5.8–6.9)	5.9 ± 1.0 5.9 (5.1–6.8)	6.4 ± 0.9 6.4 (5.9–6.9)	0.008 **

NG, nasogastric; *, *p*-value < 0.05; **, *p*-value < 0.01; ****, *p*-value < 0.0001 for the statistical significance.

**Table 3:** Univariate and multivariate logistic regression for toleration to liquids.

Variables	Univariate Logistic Regression		Multivariate Logistic Regression	
	Odds Ratio (95%CI)	*p*-Value	Odds Ratio (95%CI)	*p*-Value
Age	0.99 (0.97–1.03)	0.871	0.98 (0.93–1.04)	0.658
Sex (female vs. male)	1.37 (0.68–2.75)	0.375	1.17 (0.37–3.7)	0.788
NLR	1.39 (1.24–1.56)	<0.0001 ****	0.99 (0.77–1.26)	0.916
PLR	1.01 (1.0–1.01)	<0.0001 ****	1.00 (0.99–1.00)	0.582
LMR	0.47 (0.34–0.66)	<0.0001 ****	0.62 (0.38–1.01)	0.053
SII	1.0 (1.0–1.0)	<0.0001 ****	1.0 (1.0–1.0)	0.007 **
Stoma	1.53 (0.76–3.11)	0.236		
Multiorgan resection (yes vs. no)	4.79 (1.98–11.62)	0.001 ***	7.78 (2.01–30.11)	0.003 **
Hb	0.88 (0.76–1.01)	0.084	0.92 (0.71–1.2)	0.542
Protein	0.61 (0.42–0.89)	0.010 **	1.11 (0.6–2.05)	0.507
T stage (T3 + T4 vs. T1 + T2)	2.09 (0.7–6.19)	0.185		
N stage				
N1 vs. N0	2.39 (0.97–5.88)	0.058	4.2 (0.95–18.41)	0.058
N2 vs. N0	2.77 (1.08–7.11)	0.034 *	3.19 (0.64–15.93)	0.158
M stage (M1 vs. M0)	1.26 (0.54–2.97)	0.593		
Laparoscopic surgery	0.25 (0.06–1.1)	0.066	0.26 (0.03–1.92)	0.185
Obesity	2.24 (0.88–5.73)	0.091	4.8 (1.1–21.11)	0.038 *

*, *p*-value < 0.05; **, *p*-value < 0.01; *** *p*-value < 0.001 ****, *p*-value < 0.0001 for the statistical significance.

**Table 4:** Univariate and multivariate logistic regression for nasogastric tube reinsertion after colorectal cancer surgery.

Variables	Univariate Logistic Regression		Multivariate Logistic Regression	
	Odds Ratio (95%CI)	*p*-Value	Odds Ratio (95%CI)	*p*-Value
Age	1.01 (0.97–1.05)	0.764	0.98 (0.93–1.04)	0.591
Sex (female vs. male)	1.63 (0.73–3.64)	0.230	1.61 (0.49–5.24)	0.431
NLR	1.36 (1.22–1.51)	<0.0001 ****	1.06 (0.85–1.33)	0.852
PLR	1.01 (1.01–1.02)	<0.0001 ****	1.01 (1.00–1.01)	0.054
LMR	0.4 (0.27–0.63)	<0.0001 ****	0.55 (0.29–1.06)	0.076
SII	1.0 (1.0–1.0)	<0.0001 ****	1.0 (1.0–1.0)	0.023 *
Stoma	1.59 (0.71–3.56)	0.260		
Multiorgan resection (yes vs. no)	3.04 (1.10–8.41)	0.032 *	3.54 (0.75–16.68)	0.111
Hb	0.84 (0.72–1.0)	0.053	0.78 (0.59–1.03)	0.0781
Protein	0.55 (0.36–0.85)	0.007 **	0.85 (0.44–1.64)	0.984
T stage (T3 + T4 vs. T1 + T2)	1.38 (0.45–0.42)	0.573		
N stage				
N1 vs. N0	2.78 (1.02–7.56)	0.046 *	11.92 (1.98–71.78)	0.007 **
N2 vs. N0	1.83 (0.59–5.7)	0.299	3.11 (0.46–21.23)	0.247
M stage (M1 vs. M0)	1.3 (0.49–3.42)	0.596		
Laparoscopic surgery	0.38 (0.09–1.64)	0.193		
Obesity	2.74 (1–7.51)	0.05	12.67 (2.49–64.24)	0.002 **

*, *p*-value < 0.05; **, *p*-value < 0.01; ****, *p*-value < 0.0001 for the statistical significance.

**Table 5:** Demographics and clinical characteristics among the three I-FEED categories in colorectal cancer patients.

Characteristics	I-FEED			*p*-Value
	Normal (*n* = 207)	POGI (*n* = 30)	POGD (*n* = 23)	
Age (years)	68.1 ± 10.6 70 (62–76)	69.10 ± 9.97 71(64–76.25)	70.35 ± 9.2 71 (64–79)	0.632 ^a^
Sex, male, *n* (%)	114 (55.1%)	20 (66.7%)	10 (43.5%)	0.239 ^b^
Comorbidities	3.3 ± 2.6 3 (1–5)	3 ± 2.5 2.5 (1.75–4)	3.8 ± 3.8 2 (1–9)	0.791 ^a^
SII	832.9 ± 508.3 721.7 (491.8–1071.3)	1940.94 ± 1694.7 1677.26 (1282.5–2181.7)	4153 ± 2069.8 4119.4 (2020–6328.3)	<0.0001 ^a^
NLR	3.2 ± 1.7 2.9 (2.1–3.7)	6.88 ± 5.71 4.93 (3.31–8.52)	10.6 ± 4.7 9.2 (6.4–14.3)	<0.0001 ^a^
Hb	11.1 ± 2.3 11 (9.7–12.3)	10.9 ± 2.9 10.7 (9.3–13.5)	9.6 ± 2.3 9.3 (7.9–11.8)	0.038 ^a^
Protein	6.4 ± 0.9 6.5 (5.9–7)	6.1 ± 0.96 6.3 (5.8–6.6)	5.7 ± 1 5.5 (4.9–6.3)	0.001 ^a^
Tf (days)	3.03 ± 0.7 3 (3–3)	3.93 ± 0.64 4 (4–4)	5.26 ± 0.96 5 (5–6)	<0.0001 ^a^
Toleration liquids/48 h, yes, *n* (%)	5 (2.4%)	10 (33.3%)	22 (95.7%)	<0.0001 ^b^
NG tube reinsertion/48 h, yes, *n* (%)	0	6 (20%)	21 (91.3%)	<0.0001 ^b^
Stoma, yes, *n* (%)	69 (33.3%)	9 (30%)	12 (52.2%)	0.169 ^b^
Multiorgan resection, yes, *n* (%)	18 (8.7%)	5 (16.7%)	3 (13%)	0.35 ^b^
T-stage, *n* (%)				0.34 ^b^
T1	8 (3.9%)	1 (3.3%)	0	
T2	35 (16.9%)	3 (10%)	2 (8.7%)	
T3	104 (50.2%)	17 (56.7%)	9 (39.1%)	
T4	60 (29%)	9 (30%)	12 (52.2%)	
N-stage, *n* (%)				0.24 ^b^
0	84 (40.6%)	12 (40%)	4 (17.4%)	
1	72 (34.8%)	9 (30%)	12 (52.2%)	
2	51 (24.6%)	9 (30%)	7 (30.4%)	
M-stage, *n* (%)				0.30 ^b^
0	171 (82.6%)	25 (83.3%)	16 (69.6%)	
1	36 (17.4%)	5 (16.7%)	7 (30.4%)	

The mean ± SD, median, and IQR were used as measures of the central tendency for the assessment of the continuous variables. ^a^ The Kruskal–Wallis test was used to compare the continuous variables among the 3 groups on I-FEED. ^b^ The chi-square test was used to compare the qualitative variables among the 3 groups on I-FEED. POGI, postoperative gastrointestinal intolerance; POGD, postoperative gastrointestinal dysfunction; Tf, time to first flatus.

## Data Availability

Data are available on request.

## Abbreviations

IDR: immunocyte-derived ratio; NLR, neutrophil-to-lymphocyte ratio; SII, systemic immune-inflammation index; PLR, platelet-to-lymphocyte ratio; LMR, lymphocyte-to-monocyte ratio; Hb, hemoglobin; POI, postoperative ileus; TL, toleration for liquids; Tf, time to pass flatus; NGtR, nasogastric tube reinsertion; MR, multiorgan resection; POGI, postoperative gastrointestinal intolerance; POGD, postoperative gastrointestinal dysfunction.
